# NNK, a Tobacco-Specific Carcinogen, Inhibits the Expression of Lysyl Oxidase, a Tumor Suppressor

**DOI:** 10.3390/ijerph120100064

**Published:** 2014-12-23

**Authors:** Guang Cheng, Jianmin Li, Maoguen Zheng, Yinzhi Zhao, Jing Zhou, Wande Li

**Affiliations:** 1 The Central Lab, Hebei United University Affinity Hospital, Tangshan 063000, China; E-Mails: lijianmints@sina.com (J.L.); zmg111222@sina.com (M.Z.); 2 Department of Biochemistry, Boston University School of Medicine, Boston, MA 02118, USA; E-Mails: yinzhi@bu.edu (Y.Z.); jz@bu.edu (J.Z.); wandeli@bu.edu (W.L.)

**Keywords:** lysyl oxidase (LOX), 4-(methylnitrosamino)-1-(3-pyridyl)-1-butanone (NNK), CpG methylation, histone H3 acetylation

## Abstract

A tobacco-specific carcinogen, 4-(methylnitrosamino)-1-(3-pyridyl)-1-butanone (NNK), is believed to contribute to the cancer burden in cigarette smokers. To evaluate NNK effects on the expression of lysyl oxidase (LOX), a tumor suppressor, we examined this enzyme at various levels in NNK-treated rat fetal lung fibroblasts (RFL6). Exposure of cells to NNK reduced levels of steady-states LOX mRNA and new transcript synthesis. NNK inhibited all LOX protein species in a dose-dependent manner. Although 300 µM NNK markedly decreased the level in the 46 kDa preproenzyme, under same conditions, there was no detectable amounts of the 50 kDa proenzyme and the 32 kDa mature enzyme suggesting NNK perturbing the LOX protein processing to its mature form. Moreover, NNK also suppressed LOX activities in conditioned media of treated cells. At the promoter level, NNK enhanced methylation of CpG, but decreased acetylation of histone H3 at the core promoter region of the LOX gene. These results indicated that transcriptional and translational processes of LOX are major targets for NNK. Thus, inactivation of tumor suppressor gene LOX may play a critical role in NNK carcinogenesis.

## 1. Introduction

Lysyl oxidase (LOX) (E.C. 1. 4. 3.13), a Cu-dependent enzyme, oxidizes specific peptidyl lysine residues in collagen and elastin, and thus catalyzes the cross-linkage of these proteins essential for extracellular matrix (ECM) generation and healing [1]. Notably, LOX can also catalyze other basic proteins (pI > 8) such as basic fibroblast growth factor (bFGF), histone H1 and H2, *etc.* [2,3,4]. This enzyme has been found within the cell nucleus, where it may modulate the chromatin packing state [5,6]. LOX is considered as a tumor suppressor gene as evidenced by that expression of transfected LOX cDNA suppressed Ha-*ras*-induced cell transformation indicating a *ras*-suppressor effect of LOX [7]. Recently, high levels of LOX have been detected in some tumors under hypoxia conditions facilitating tumor metastasis [8]. Thus, LOX may play multiple roles in biology.

A tobacco-specific nicotine-nitrosated derivative, 4-(methylnitrosamino)-1-(3-pyridyl)-1-butanone (NNK), has been demonstrated as a very potent carcinogen in rodents, particularly in rats [9]. One current “full-flavored cigarette” contains 131 ng NNK. The life time exposure of NNK to a smoker is about 1.1 mg/kg close to the tumorigenic dose of NNK for rats (1.8 mg/kg). NNK prefers to induce lung adenoma and adenocarcinoma unrelated with the administration route [10]. In human body, NNK is activated by cytochrome P-450 (CYP) to exhibit its carcinogenicity [11]. Although NNK is known to induce DNA adducts and gene mutations [12], the precise molecular mechanisms for NNK pathogenesis and carcinogenesis remain to be understood.

To further understand mechanisms for NNK carcinogenicity, we have examined NNK effects on the expression of LOX, a tumor suppressor gene. Results showed NNK down-regulation of LOX in treated rat lung fibroblasts at such multiple levels as DNA (promoter), mRNA, protein and catalytic activity. NNK inhibited LOX promoter activities as a result of enhancement of CpG methylation and reduction of histone H3 acetylation at the core promoter region of the LOX gene.

## 2. Experimental Section

### 2.1. Materials

NNK with 98% purity was purchased from Toronto Research Chemicals (North York, ON, Canada). Diaminopentane and horseradish peroxidase (HRP) were from Sigma-Aldrich Co. (St. Louis, MO, USA). Amplex red was from Life Tech. (Grand Island, NY, USA). Rabbit anti-LOX antibody was developed by Wande Li’s Lab (Boston Univ. Sch. Med. Boston, MA. USA) in cooperation with Rockland Immunochem. Inc. (Gilbertsville, PA, USA). Mouse anti-RNA polymerase II (RNA-PolyII), acetylated histone H3, and glyceradehyde 3-phosphate dehydrogenase (GAPDH) were from Santa Cruz Biotech. (Santa Cruz, CA, USA). [α-^32^P]UTP was from PerkinElmer (Boston, MA, USA). Synthetic oligonucleotide primers used for the PCR were purchased from Integrated DNA Technologies (Coralville, IA, USA). All tissue culture products were from Invitrogen Co. (Carlsbad, CA, USA).

### 2.2. Cell Culture and NNK Exposure

The rat fetal lung fibroblasts (RFL6) obtained from ATCC were cultured in Dulbecco’s modified Eagle’s medium (DMEM) supplemented with 10% fetal bovine serum (FBS) at 37 °C in a 5% CO_2_ and 95% air incubator as previously described [13]. Stock cultures were derived from the frozen cell line and passaged every four days in a total of six passages. To obtain growth-arrested cultures, cells were incubated in 0.3% FBS/DMEM for three days, changed to fresh medium and used for experiments [13]. NNK was dissolved in DMSO as a stoke solution. To identify effects of NNK on cell phenotype changes, growth-arrested cells were exposed to final concentrations of NNK at 10, 30, 100, and 300 µM, respectively, for 48 h. Cell viability was determined by the trypan blue exclusion test. Control and NNK-treated cells in triplicate dishes were trypsinized, washed and stained with 0.4% trypan blue (Gibco, Rockville, MD, USA). The number of viable (non-stained) and dead (stained) cells were counted using a hemocytometer. Note, this dose/time range or above this dose/time range has been used to identify DNA damage and other phenotype changes in cultured human white blood cells [14] and in ARPE 19 cells [15] in response to NNK. Control cells were exposed to vehicle only.

### 2.3. Assay for LOX Activities

Fluorometric assays for H_2_O_2_ release in the LOX-substrate reaction were carried out to assess NNK effects on LOX catalytic activities in the cell model using diaminopentane as a substrate and Amplex red as a hydrogen peroxide probe as described [16]. In a typical assay, samples (e.g., 500 µL conditioned medium) were mixed with the reaction mixture containing 0.05 M sodium borate, pH 8.2, 10 mM diaminopentane, 10 µM Amplex red, 40 µg HRP, and 2 M urea in a final volume 2 mL in the presence or absence of 0.5 mM β-aminopropionitrile (BAPN), an active site inhibitor of LOX. All enzyme activities were continuously monitored for at least 300 s at excitation and emission wavelengths of 563 and 587 nm, respectively, at a constant temperature of 37 °C, as specified in the thermostatted cuvette chamber of an LS 55 Luminescence Spectrometer (PerkinElmer Instruments, Shelton, CT, USA). Results were expressed as fluorescence values at 300 s after the reaction, corrected for background levels of H_2_O_2_ release determined in the reaction mixture supplemented with BAPN, and normalized to total cell protein.

### 2.4. Western Blot Analysis

Control and treated cells were lysed in the RIPA buffer composed of 1 × PBS, 1% NP-40, 0.5% sodium deoxycholate, 0.1% SDS, and 2 M urea, pH 7.4, and the protease inhibitor cocktail (Roche, Mannheim, Germany). After microcentrifugation, protein concentrations in supernatants were determined by the BCA protein assay reagents (Pierce, Rockford, IL, USA). Cell lysates containing equal amounts of protein (25 or 50 µg) were boiled in an SDS sample buffer and analyzed by SDS-PAGE. The separated proteins in the gel were then transferred to a nitrocellulose membrane (Schleicher & Schuell, Keene, NH, USA). Nonspecific binding sites were blocked by incubating the nitrocellulose membrane in Tris-buffered saline containing 0.1% Tween-20 with 5% nonfat dry milk. Membranes were then incubated overnight at 4 °C with primary antibody such as a rabbit anti-LOX (1:1000) or mouse anti-tubulin (1:1000). After washing, membranes were then incubated with the corresponding secondary antibody (*i.e.*, anti-rabbit or anti-mouse IgG) conjugated with HRP (1:2000, Santa Cruz Biotech) for 1 h at room temperature. Blots were developed with an enhanced chemiluminescence system (PerkinElmer Life Sciences, Boston, MA, USA) and molecular weights determined by comparison with BenchMark prestained protein ladder (Invitrogen). Protein bands were quantitated by the 1D Scan EX software (Scan Analytics, Fairfax, VA, USA) as described [13,16]. Experiments as shown here and below were repeated at least three times with reproducible results, and a representative one is presented, unless otherwise indicated.

### 2.5. Reverse Transcription (RT) and Quantitative Real-Time PCR Analysis

Total RNA was extracted from control and treated cells using Trizol reagent (Invitrogen). The first-strand cDNA was synthesized with 1 µg of the total RNA using the SuperScript first-strand synthesis system for RT-PCR (Invitrogen). Using one-twentieth of the cDNAs as templates, the PCR was carried out under conditions as described [17]: denaturation at 94 °C for 30 s and annealing at 65 °C for 30 s, followed extension at 72 °C for 3 min. The primer pairs were: forward (F), 5’-GATGGATCCTCTAGAATGCGTTTCGCCTGGACCGTGCTCTTTCTGGG-3’ and reverse (R), 5’-GATCTCGAGGATATCCTAATACGGTGAAATGGTGCAGCCTGAGGCATAGGC-3’ for LOX; and F, 5’-GACTCTACCCACGGCAA-3’ and R, 5’-GGATGACCTTGCCCACA-3’ for GAPDH, an internal control. PCR products were analyzed on a 2.2% agarose gel, stained with ethidium bromide and visualized on a UV transilluminator. PCR-amplified DNA bands were quantitated by the 1D Scan software as described [17].

The real-time PCR was performed in a GeneAmpR 5700 Sequence Detection System (SDS) using a MicroAmp optical 96-well reaction plate with optical caps (PE Applied Biosystems). Primers and TaqMan probes used were: F, 5’-CAGGCACCGACCTGGATATGGCACC-3’ and R, 5’-GTACGTACGTGGATGCCTGGATGTAG-3’ for LOX; F, 5’-ATGACTCTACCCACGGCAAG-3’ and R, 5’-TACTCAGCACCAGCATCACC-3’ for GAPDH. The TaqMan probe for LOX was 5’ 6FAM-AGTACGGTCTCCCGGACCTGG TAC-TAMRA 3’ and the probe for GAPDH was 5’ VIC –AGCTGGTCATCAACGGGAAACCCATCA-TAMRA 3’. The reaction mixture (50 μL) contained 2× TaqMan Universal PCR Master Mix (PE Applied Biosystems), 20 pM of sense and antisense primers, 10 pM of TaqMan probe and 5 μL of cDNA mixture synthesized from RNA by reverse transcription using the SuperScript first-strand synthesis system. Thermocycling program was 40 cycles of 95 °C for 15 s and 60 °C for 1 min with an initial cycle of 95 °C for 10 min. At each cycle, PCR products were monitored by observation of changes in fluorescence of the reporter dye from the TaqMan probes. After the PCR, a melting curve was constructed in the range of 60 °C to 95 °C. All data were analyzed using the GeneAmp 5700 SDS software.

### 2.6. The Nuclear Run-On Assay

Relative rates of LOX transcription in control and treated cells were evaluated by the nuclear run-on assay as described [18]. Cell pellets were gently resuspended in a nuclear isolation buffer and incubated on ice with intermittent microscopic examination for nuclear integrity. The nuclei were centrifuged at 500× g and resuspended in a nuclear freezing buffer either for direct use or for storage in liquid nitrogen. For the nuclear run-on reaction, 100 μL of thawed nuclei were mixed with 30 μL of a 5× run-on buffer with NTP containing 100 μCi [α-^32^P]UTP and 5 μL of the Sarkosyl stock to give a final concentration of 0.06%. The mixture was incubated for 30 min at 30 °C, then 15 μL of DNase I (1 U/μL) were added and the incubation continued for another 15 min. RNA was isolated by a single step Trizol extraction and the incorporation of ^32^P determined by γ–counting. Plasmids containing LOX cDNA and GAPDH cDNA were slot-blotted onto the nitrocellulose membrane using a BioDot SF apparatus (BioRad, Hercules, CA, USA). The blots were prehybridized in 1% SDS/10% dextran sulfate, 1.4 M NaCl and 325 μg/mL each of herring sperm DNA and yeast tRNA for 2 h at 60 °C followed by treatment with RNasin plus DTT. Radiolabeled RNAs were hybridized onto filters for 2 days. The filters were then washed, dried and autoradiographed on preflashed film. The densities of labeled RNA bands on the film were analyzed by the 1D Scan software as described [17].

### 2.7. Cell Transfection and Assays for Reporter Gene Products

To probe regulation of LOX transcription we have created various LOX promoter-reporter constructs [19]. Since the LOX promoter fragment from −804 to −1 (relative to ATG) (Prom-804) exhibited the maximal luciferase activity in transfected RFL6 cells, this construct was used as a model for assessing effects of NNK on the LOX promoter activation. Cells were plated at 5 × 10^5^ cells per 60 mm dish containing 5 mL of 10% FBS/DMEM. After 24 h incubation, cells were co-transfected with the LOX promoter-luciferase construct (2 µg) as well as the pRL-TK vector, an internal control (0.5 μg, Promega, Madison, WI, USA), by using lipofectamine reagent (Invitrogen) as described [19]. Note that cells co-transfected with pGL3-basic vectors containing the luciferase gene without the LOX promoter and the pRL-TK vector were always included in any experiments to evaluate the background. Following 6 h posttransfection incubation, cells were incubated in 10% FBS/DMEM for an additional 18 h-period, washed, and then exposed to NNK for 48 h at indicated doses. Luciferase activities in cell lysates extracted from control and NNK-treated cells were measured by luminometry as recommended by supplier (Promega). Firefly luciferase activities elicited by the LOX promoter were normalized to Renilla luciferase activities derived from the pRL-TK vector and expressed as relative luciferase activities as instructed by manufacturer (Promega).

### 2.8. Assay for Methylation of the LOX Core Promoter Region 

Genomic DNA from control and treated cells was isolated using the DNA Mini Preparation kit (Qiagen, Inc., Valencia, CA, USA). PCR assays were performed by using the promoter methylation PCR kit (Panomics, Redwood City, CA, USA) as described [20]. Briefly, 2 μg of genomic DNA were digested with 10 units Mse I (New England Biolabs, Boston, MA, USA) to produce small fragments of DNA, which retain the CpG islands. Following incubation with methylation binding protein (MBP) to form a protein/DNA complex, methylated DNA was isolated by centrifugation using a separation column and amplified at the following PCR program: 94 °C for 5 min, 94 °C for 1 min, 56 °C for 1 min, and 72 °C for 2 min for 35 cycles. PCR products were analyzed on 2.2% agarose gel. The primer pair F, 5’-TTCAGACACTGTGCGCTCTC-3’ and R, 5’-AGGAGGGAGACCTCTTCGAG-3’ was used for amplification of the methylated LOX fragment on the promoter region (205 bp) containing 15 CpG islands.

### 2.9. Chromatin Immunoprecipitation (ChIP) Assay 

To determine transcription factor binding to the LOX promoter region, the ChIP assay was performed as described [19] with the EpiQuik Chromatin Immunoprecipitation Kit based on the protocol provided by the supplier (Epigentek Group Inc., Brooklyn, NY, USA). Cellular components were cross-linked by incubation of control and treated cells at the same number (2 × 10^6^) with 1% formaldehyde at room temperature for 10 min. The cross-linking reaction was stopped by addition of glycine to a final concentration of 125 mM. Nuclei were extracted with a nuclear isolation buffer, resuspended in a nuclear lysis buffer with protease inhibitor cocktail and then sonicated to shear DNA to lengths between 200 and 1000 bp. After centrifugation, cell debris was discarded and DNA containing supernatants were diluted with the ChIP dilution buffer and aliquots of samples were removed out. Diluted DNA samples were transferred into the strip wells that were precoated with the monoclonal antibodies against rat RNA-PolyII, and acetylated histone H3 (Santa Cruz Biotech.), and incubated at room temperature for 90 min with shaking. After successively washing with the washing buffer and finally with the TE buffer (10 mM Tris-HCl, pH 8.0, 1 mM EDTA), precipitated DNA-protein complex samples were treated with proteinase K (250 µg/mL) in the DNA release buffer for 15 min and then incubated in the reverse buffer for 90 min at 65 °C. The DNA samples were collected by the P-spin columns, washed with 70% and 90% ethanol successively, and then eluted with the elution buffer. Using purified DNA as a template, PCR was conducted under the following conditions: initial denaturation at 94 °C for 2 min, 30 cycles each with denaturation at 94 °C for 30 s, annealing at 55 °C for 30 s and extension at 72 °C for 1 min, and final extension at 72 °C for 5 min. Primers were used in ChIP assays as follows: F, 5’-GAAGAGGTCTCCCTCCTTCG-3’ and R, 5’-ACTGCAGCTGTCCCAGAAAG-3’ for amplifying the acetylated histone H3-bound LOX core promoter region (136 bp); F, 5’-GATGTTAGCGGGATCTCGCTCCTG-3’ and R, 5’-GTTCAACGGCACAGTCAAGGCTGAG-3’ for amplifying the RNA-PolyII binding region in the GAPDH promoter (90 bp), an internal control. PCR products were analyzed on a 2.2% agarose gel, stained with ethidium bromide and visualized on a UV transilluminator. PCR-amplified DNA bands were scanned for the density measurement as described [19].

## 3. Results and Discussion

### 3.1. NNK Effects on LOX Expression at Catalytic and Protein Levels

LOX catalyzes the post-translational modification of elastin, collagen and histone H1 by oxidizing selected lysine residues within these proteins to peptidyl α-aminoadipic-δ-semialdehyde. Subsequent spontaneous reactions of the peptidyl aldehydes yield covalent cross-linkages stabilizing the extracellular matrix and cell nucleus [1]. LOX is synthesized as a 46-kDa preproenzyme by fibrogenic cells. After signal peptide cleavage and N-glycosylation, the resulting 50-kDa N-glycosylated proenzyme is secreted [13,16] and proteolytically processed in the extracellular space to a 32 kDa mature enzyme [16,21]. To assess NNK effects on changes in functionality of LOX, we examined catalytic activities and protein expression of this enzyme in control and treated cells.

#### 3.1.1. NNK Inhibition of LOX Catalytic Activity in NNK Treated Cells

**Figure 1:**
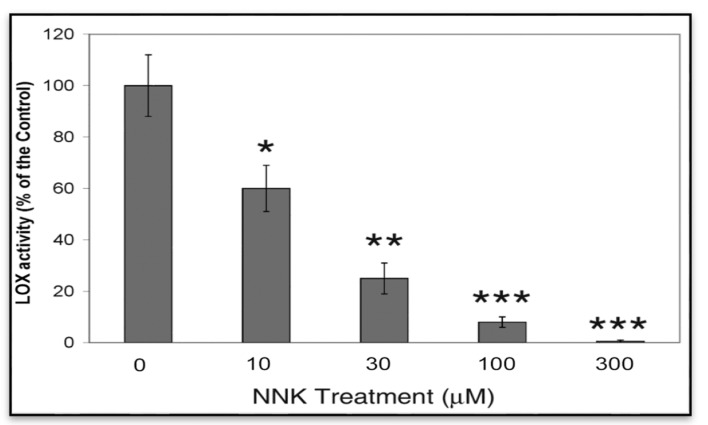
NNK inhibition of LOX activities in treated cells. Growth-arrested RFL6 cells were treated with NNK at 0–300 µM for 48 h. LOX catalytic levels were measured in the conditioned media with the fluorometric assay as described in the Experimental. Data shown are the mean ± SD (*n* = 3). * *p* ≤ 0.05, ** *p* ≤ 0.01, *** *p* ≤ 0.001 compared with the control.

Since LOX is a secreted protein its activity was mainly present in the ECM. To assess LOX catalytic expression, conditioned media from control and treated cells were collected for activity assays as described [13,16]. LOX activities in cell conditioned media were probed by the H_2_O_2_ release assay using diaminopentane as a substrate and Amplex red as a hydrogen peroxide probe. As shown in Figure 1, treated cells displayed a dose-dependent inhibition of LOX activities in conditioned media amounting to 61, 23 and 8 and 2% of the control, respectively, for cells treated for 48 h with 10, 30, 100 and 300 µM NNK. Notably, as determined by the trypan blue exclusive assay, 96.0 ± 6.0, 94.0 ± 9.0, 95.0 ± 8.0, 90 ± 11.0, and 88.0 ± 12.0% of growth-arrested cells remained viable following incubation for 48 h in the presence of 0, 10, 30, 100 and 300 µM of NNK, respectively. Thus, the observed changes in LOX activities in NNK-treated cells were unlikely resulted from effects of cell death.

#### 3.1.2. NNK Inhibition LOX Synthesis and Processing in Treated Cells

**Figure 2:**
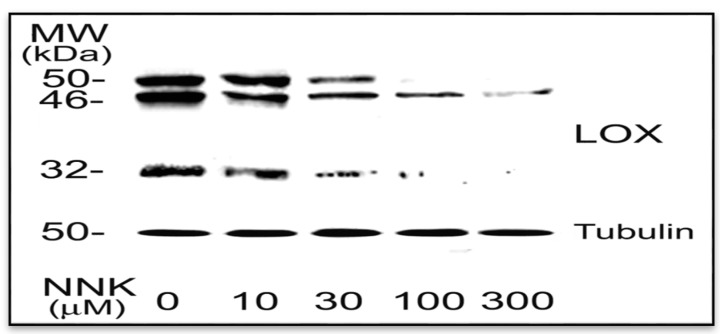
NNK inhibition of LOX protein profile in treated cells. Growth-arrested RFL6 cells were treated with NNK at 0–300 µM for 48 h. Total cell proteins were extracted and aliquots of protein samples (25 µg each) were analyzed on SDS-PAGE and detected by Western blot and densitometry measurement. The 46-, 50- and 32- kDa proteins are LOX species, the bottom protein is tubulin with 50 kDa, an internal control. Experiments were repeated three times, one of which is presented here.

Western blot was performed to identify NNK effects on the LOX protein profile. As shown in Figure 2, LOX antibody immunoreactive proteins in RFL6 cell extracts include a 46-, a 50-, and a 32-kDa bands representing a typical protein profile of LOX synthesis and processing by fibrogenic cells including the 46-kDa preproenzyme, the 50 kDa proenzyme and the 32-kDa functional species [13,21]. Since a part of the mature enzyme was attached to the cell membrane and the ECM, the 32-kDa protein was positively detected in the cell extract fraction. Comparatively, NNK treated cells exhibited markedly decreased levels in the 46-, the 50-, and the 32-kDa proteins. The densitometry analysis indicated that the 46-kDa preproenzyme was reduced to 60.5, 48.0, 30.0 and 14.3% of the control; the 50-kDa proenzyme decreased to 70.0, 38.5, 0.2 and 0% of the control; and the 32-kDa mature enzyme declined to 69.0, 31.0, 8.0 and 0.1% of the control; respectively in cells treated with 10, 30, 100 and 300 µM NNK for 48 h. Notably, the 50 kDa and the 32 kDa species of LOX were more sensitive to NNK in treated cells. Although 300 µM NNK, markedly decreased level in the 46 kDa preproenzyme, under same conditions, there almost was no detectable amount of the 50 kDa proenzyme and the 32 kDa mature enzyme. In contrast, neither control nor treated cells were found significant changes in expressions of tubulin protein, an internal control. These results suggest that NNK not only inhibited LOX synthesis but also perturbed the LOX processing to form its mature species.

### 3.2. NNK Effects on LOX Transcriptional Levels

Transcription is a process of nucleoside triphosphate polymerization into RNA in a DNA-template-dependent manner [22]. The synthesized massager RNA with genetic information from DNA is processed and trans-located from the nucleus into the ribosome in the endoplasmic reticulum (ER), where they are translated into a polymer of amino acids, a protein. To further define NNK modulation of LOX transcription, we directly compared measurements of the steady-state mRNA levels and the relative mRNA synthesis rate of LOX in control and treated cells.

#### 3.2.1. NNK Inhibition of the Steady-State mRNA Levels of LOX in Treated Cells

**Figure 3:**
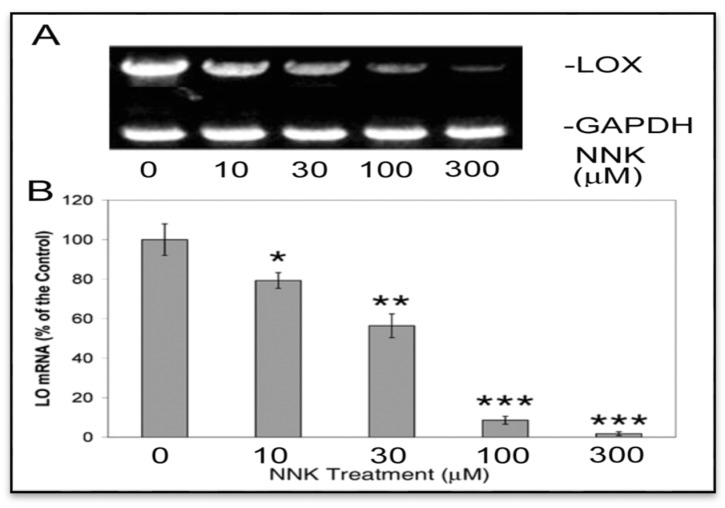
NNK inhibition of LOX steady–state mRNA levels in treated cells as revealed by reverse transcription (RT)-PCR and agarose gel electrophoresis (**A**) and quantitative real-time-PCR (**B**). (**A**) Total RNA (1 µg) was extracted from growth-arrested control and treated cells using Trizol reagent. Reverse-transcription cDNA was produced using the SuperScript first-strand synthesis system. LOX and GAPDH (an internal control) cDNA fragments were amplified by PCR and analyzed on a 2.2% agarose gel. Densities of PCR-amplified gene fragments on the gel as described here and below were measured with the 1D Scan software. (**B**) The real-time PCR was performed by the GeneAmpR 5700 Sequence Detection System (SDS) using reverse-transcription DNA as a template. PCR products were monitored by fluorescence from the TaqMan probes for LOX and GAPDH (an internal control) and analyzed using the GeneAmp 5700 SDS software. Data shown are the mean ± SD (*n* = 3). * *p* ≤ 0.05, ** *p* ≤ 0.01, *** *p* ≤ 0.001 compared with the control.

To assess LOX mRNA expression by the reverse transcription (RT)-PCR, equal amounts of total RNA isolated from growth arrested control and treated cells were added to the RT reaction mixture. Total cDNA produced by the RT reaction and PCR amplification was evaluated as levels of transcripts [17]. As shown (Figure 3A), cells exposed to NNK exhibited dose-dependent decreases in levels of LOX cDNA (1.3 kb) in comparison to the internal control, GAPDH cDNA (500 bp). OneD Scan EX analysis revealed that NNK at 10, 30, and 100 and 300 µM reduced LOX cDNA levels to 71, 58, 37, and 16%, respectively, of the control without NNK treatment. Furthermore, quantitative real-time PCR indicated that LOX mRNA levels were decreased to 80 ± 6, 56 ± 4 and 12 ± 2 and 2 ± 1% of the control in cells treated with 10, 30, 100 and 300 µM NNK, respectively (Figure 3B). These results illustrated NNK inhibition of LOX steady-state mRNA expression.

#### 3.2.2. NNK Inhibition the Initial Transcription Rate of LOX in Treated Cells 

**Figure 4:**
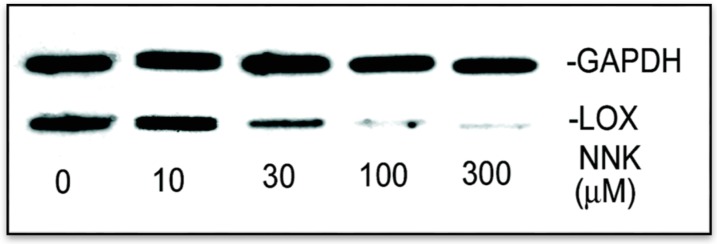
Reduction of the relative transcription rate of LOX in NNK treated cells revealed by the nuclear run-on assay. Nuclei were freshly isolated from growth-arrested control and treated cells under the same conditions as described in the Experimental. Nascent transcripts were labeled with ^32^P-UTP and hybridized to a previously prepared filter containing cDNAs for LOX and GAPDH (an internal control). Hybridized radiolabeled RNAs onto filters were washed, dried and autoradiographed on preflashed film. The densities of labeled RNA bands on the film were analyzed by the 1D Scan software.

The steady-state mRNA levels as determined by either the RT- PCR or the real-time PCR (Figure 3) actually reflect a composite of both the synthesis and the degradation rates of mRNA [18]. To identify NNK effects on upstream transcriptional initiation of LOX, the nuclear run-on assay was carried out [18]. As shown in Figure 4, using the internal control GAPDH as reference, levels of [^32^P-UTP]-labeled transcripts hybridized to the LOX cDNA were markedly diminished in nuclei of NNK-exposed cells, amounting to 93, 41, 11, and 5% of the RFL6 control, respectively, in cells treated with 10, 30, 100, and 300 µM NNK. Thus, reduction of new transcript synthesis by NNK is a critical mechanism for down-regulation of LOX mRNA in treated cells.

### 3.3. NNK Effects on the LOX Promoter Activation 

In eucaryotic cells, the transcription is regulated by the gene promoter located at the 5’-flanking region of a gene. To investigate the regulation of the LOX gene transcription, we have cloned the rat LOX promoter and identified its core promoter and transcription start sites [19]. The core promoter offen sequenced by the TATA box is a site for the action of the RNA-PolyII transcriptional machinery playing a central role in gene transactivation. RNA-PolyII along with auxiliary transcription factors (TFs), binds to the core promoter and catalyzes the synthesis of mRNA from the DNA template [23,24]. The cloned rat LOX promoter −804/−1 (relative to ATG) with the maximal activity appears to contain an Inr-DPE core promoter, free of the typical TATA box. Rat LOX transcriptions are started at multiple sites from −78 to −51 relative to ATG, one of which is the adenosine residue overlaping with the INR element (5’-TCATTTTT-3’) located from −53 to −46 in the rat LOX promoter [19]. Furthermore, the DPE sequence 5’-GGACG-3’ from −18 to −14 is mapped approximately 30 bp after the adenosine residue in the Inr motif [19]. Generally, the Inr and the DPE coordinately work as a single core promoter unit for the gene transcription [25]. Notably, transcription is a multi-step process involving distinct chromatin modifying and remodeling that control the proper recruitment of TFs and assembly of the RNA-PolyII pre-initiation complex [26]. Acetylation of histone N-terminal lysines is intimately linked to chromatin remodeling for transcription regulation. Reversible acetylation of histones such as H3 facilitates access of transcriptional machinery to DNA. Thus, acetylated histone H3 is a marker for special gene activation [27]. Here, we further identified NNK effects on LOX promoter activities and assessed acetylated histone H3 binding to the LOX Inr-DPE region in cells exposure to NNK.

#### 3.3.1. Inhibition of LOX Promoter Activities in NNK Treated Cells

**Figure 5:**
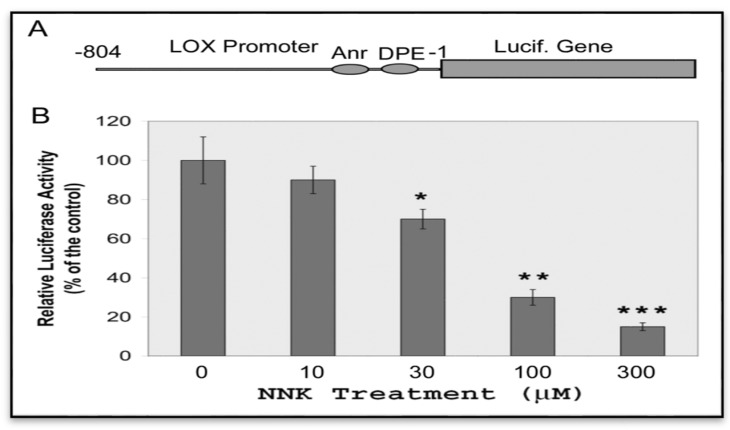
Inhibition of LOX promoter activities in NNK-treated cells. (**A**) Schematic representation of LOX promoter-reporter chimera with the Inr-DPE core promoter. (**B**) NNK inhibition of LOX promoter activities in transfected cells. RFL6 cells were transiently co-transfected with the Prom-804 construct and the pRL-TK vector, an internal control, then treated with NNK at indicated concentrations for 48 h. Luciferase activities in cell lysates were measured by luminometry. Firefly luciferase activities elicited by the LOX promoter were normalized to Renilla luciferase activities derived from the pRL-TK vector and expressed as relative luciferase activities as instructed by manufacturer (Promega). Data shown are the mean ± SD (*n* = 3). * *p* ≤ 0.05, ** *p* ≤ 0.01, *** *p* ≤ 0.001 compared with the control (100%).

We have cloned rat LOX promoter region −804/−1(Prom-804, relative to ATG) into the reporter gene construct pGL3-Basic inducing the maximal expression of the luciferase gene expression in transfected cells [19]. Thus, this LOX promoter-reporter gene construct (Figure 5A) was used for assessing effects of NNK on the LOX promoter activation. RFL6 cells were transiently co-transfected with the Prom-804 construct and pRL-TK vector, an internal control. Firefly luciferase activities elicited by the LOX promoter were normalized to Renilla luciferase activities derived from the pRL-TK vector and expressed as relative luciferase activities as described [19]. As shown in Figure 5B, NNK decreased LOX promoter activities in a dose-dependent manner as evidenced by that the reporter gene expressions were inhibited by 9 ± 8, 32 ± 6, 69 ± 3 and 82 ± 1%, respectively, in cells exposed to NNK at 10, 30, 100 and 300 µM. Thus, NNK as a strong repressor inhibited LOX gene transactivation.

#### 3.3.2. Inactivation of the Core Promoter of the LOX Gene in NNK-Treated Cells

**Figure 6:**
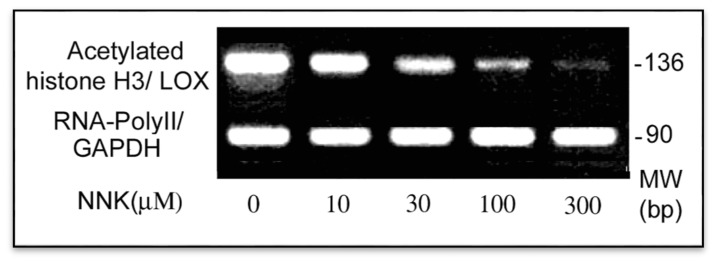
Inactivation of the LOX core promoter in NNK treated cells. ChIP and PCR assays were performed to elucidate the active status of the LOX core promoter in treated cells by assessing acetylated histone H3 binding to the core promoter region. DNAs were isolated from control and NNK treated cells each with 2 × 10^6^, sonicated and immunoprecipitated with an antibody against acetylated histone H3 or RNA-PolyII. Using immunoprecipitated DNA as a template, the PCR with primer pairs as shown under Methods amplified the acetylated histone H3-bound LOX core promoter region with 136 bp, and the RNA-Poly II bound fragment of the GAPDH promoter (an internal control) with 90 bp, respectively. PCR products were analyzed on 2.2% agarose gels and densities of DNA bands measured by the 1D Scan software.

To elucidate the active status of the LOX core promoter in response to NNK, quantitation of acetylated histone H3 at the LOX Inr-DPE region was performed by the ChIP assay [19]. The anti-diacetylated histone H3 antibody was used to precipitate DNA fragments isolated from control and NNK-treated cells. Using primers as described, the PCR amplified a 136 bp fragment (−95/+41, relative to ATG) containing the LOX core promoter from −53 to −14 and an intact transcription start site cluster from −78 to −51 [19]. As shown in Figure 6, in comparison to the internal control, the GAPDH fragment bound with the RNA-PolyII, the histone H3 acetylated at the tested region of the LOX promoter in NNK-treated cells was reduced to 74, 41, 11 and 5% of the control for cells treated with 10, 30, 100 and 300 µM NNK, respectively. These results indicated NNK inactivation of the LOX core promoter as a key mechanism for down-regulation of this enzyme at the promoter level.

### 3.4. NNK Effects on Methylation of the LOX Promoter

Modification of DNA may be a key mechanism for interfering with the DNA-protein interaction. In mammals, almost 60%–90% of all CpGs are methylated [28]. Unmethylated CpGs called CpG islands are often clustered in the gene promoter regions. Abnormal methylation of CpGs found in cancers can be inherited by daughter cells after cell division inducing permanent gene transcriptional silencing [29,30]. Aberrant methylation of genomic DNAs and enhanced activities of DNA methyltransferase (DNA MeTase) were found in NNK-exposed human and animals [31,32]. Inactivation of the LOX gene by DNA methylation was reported in human gastric cancers [33]. The rat LOX promoter −804/−1 contains approximately 38 CpG dinucleotides, of which some overlap with *cis*-elements, e.g., the core promoter, MREs, HREs, *etc.* [19] To answer the question whether NNK down-regulation of LOX mRNA is due to methylation of CpGs in the promoter region, we examined the methylation status of the LOX gene promoter in cells exposed to NNK.

#### NNK Enhancement of LOX Promoter Methylation in Treated Cells

**Figure 7:**
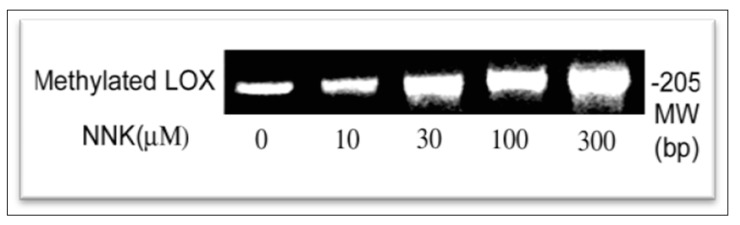
Enhancement of methylation at the LOX promoter region in NNK-treated cells as determined by using the methylation promoter PCR kit. The same amount of genomic DNA isolated from control and NNK-treated cells were digested with restriction enzyme Mse I, then incubated with MBP to form a protein/DNA complex. Methylated DNA was isolated using a separation column and amplified by PCR. PCR products as a 205 bp DNA were analyzed on 2.2% agarose gels and densities of DNA bands measured by the 1D Scan software. One typical gel among three repeated experiments is presented.

Methylated DNA fragments isolated from control and NNK-treated cells were amplified by using the promoter methylation PCR kit (Panomics, Redwood City, CA, USA) as described [20]. PCR-product encompasses the LOX gene promoter region from −279 to −75 relative to ATG containing 15 CpG. A transcription start site and several *cis*-elements such as the MRE and HRE are included in this region [19]. As shown in Figure 7, cells exposed to NNK exhibited increased methylation in the promoter region reaching 1.2, 2.4, 2.6 and 3.4-fold of the control. Thus, aberrant DNA hypermethylation existed in the LOX promoter region in cells treated with NNK.

### 3.5. LOX, a Tumor Suppressor

LOX was considered as a tumor suppressor based on the finding that cloning of mouse *ras* recission gene (rrg) cDNA revealed its sequence nearly identical with the rat LOX cDNA (>96%) [7]. Expression of transfected LOX cDNA suppressed Ha-*ras*-induced cell transformation and altered chromatin packing in the nuclei [6]. Repression of Bcl2 by the tumor suppressor activity of the LOX propeptide inhibited transformed phenotype of lung and pancreatic cancer cells [34]. Consistent with the anti-tumorigenic function of LOX, transfection of the LOX antisense into normal rat kidney fibroblasts induced anchorage-independent growth and elevation of p21-*ras* expression [35]. A variety of spontaneous human cancers displayed low levels of LOX transcription [36] such as bronchogenic carcinoma [37], gastric cancers [33], head and neck squamous cell carcinoma [38], *etc.* LOX mRNA levels were progressively declined in malignant prostate tumors either at primary or at metastatic lesions [39]. In breast tumors, LOX was down-regulated in late stromal reactions and undetectable in the loose scirrhousstroma of invading ductal carcinomas [40,41], but up-regulated in hypoxia-induced metastasis of breast cancers [8]. LOX was defect in basal and squamous cell carcinoma and its knockout led to invasion of a skin equivalent model [42]. Inactivation of the LOX gene was detected in human gastric cancers as results of DNA methylation and loss of heterozygosity [33]. Inhibition of LOX expression by somatic gene mutation was found in human colorectal tumors [43]. Our previous studies have demonstrated that bFGF is a substrate of LOX. Oxidation of bFGF by LOX blocked the proliferation of bFGF-stimulated cells and bFGF-autocrine transformed cells with highly tumorigenic potential [4]. Interestingly, LOX and its oxidized substrates exist within the nuclei of cultured vascular smooth muscle cells (VSMC) and 3T3 fibroblasts [5]. Histone H1, a critical nuclear structural protein, has been identified as a substrate of LOX in assays *in vitro* and in cells [2,3]. Apparently, LOX tumor suppressor activities are expressed by means of (1) inhibition of oncogenes such as Ha-*ras*, Bcl2, *etc*.; (2) inactivation of growth factors such as bFGF; and (3) stabilization of the nuclear structure such as oxidation of histone H1.

### 3.6. NNK, a Genetic and Epigenetic Carcinogen

NNK is naturally formed from nicotine by a nitrosation reaction occurring during the curing and processing of tobacco [9]. Once it is activated by enzymes of the cytochrome pigment (CYP) multigene family in the body [11], NNK and its metabolites can directly attach the DNA inducing genetic damages in the target organ. NNK induced p53, lacZ, cII, K-*ras*, *etc.*, gene mutation [12,44,45], The gains or losses at the chromosomes 6, 8, 11 and 14 were often found in NNK-induced tumors. Changes in the chromosomes 8, 11, 12, and 14 were positively related to the chromosome instability [46,47]. NNK induced gene polymorphisms and chromosomal instabilities are involved in cell growth, proliferation and differentiation critical for tumor initiation. Furthermore, NNK as an epigenetic carcinogen triggers a cascade of signaling pathways, resulting in uncontroling cell proliferation by growth signal self-sufficiency, apoptosis evasion, antigrowth signal insensitivity, angiogenesis sustaining, invasion and metastasis potential, and limitless replication [48]. NNK binds to nicotinic acetylcholine receptors (nAChR), especially for α7 nAChR [49,50]. It enhanced lung cancer cell proliferation by activation of the pathway of α7 nAChR in association with signal proteins such as PKC, RAF1, AKT, ERK1/2, and transcription factors such as JUN, FOS, and MYC [51,52,53]. In addition, NNK might also directly or indirectly activate other receptors such as β-adrenoceptors (β-AR), EGFR, or insulin-like growth factor receptor (IGFR) [54,55,56]. Via activations of nAChR and β-AR, NNK exhibited mitogenic properties *via* enhancement of cyclin D1 expression and G1/S transition [53,57]. NNK inhibition of apoptosis and promotion of proliferation in human bronchial epithelium cells were mediated by activation of α3/α4 nAChR followed by upregulation of AKT, MAPK, and PKC pathways [52]. In addition, NNK can also prevent cell apoptosis by modulating the anti-apoptotic Bcl2 and c-Myc proteins [58]. A loss of E-cadherin is a major pathologic event in epithelial to mesenchymal transition (EMT) critical for cancer metastasis. NNK enhanced colon cancer cell migration by down-regulation of E-cadherin [49]. NNK induces DNA methyltransferase 1 accumulation and hypermethylation of tumor suppressor genes such as p16 (cyclin-dependent kinase inhibitor 2A, multiple tumor suppressor 1), death-associated protein kinase (DAPK), *etc.* [59,60]. In this study, we reported DNA methylation by NNK readily existing in the promoter of the LOX gene.

## 4. Conclusions

Data presented in this study demonstrated that NNK inhibition of LOX at DNA (promoter), mRNA, protein and catalytic levels. Enhancement of promoter methylation, inhibition of core promoter activity, repressing of new mRNA initiation, reduction of steady-state mRNA levels, blockage of processing of the preproenzyme to the mature enzyme and abolishment of catalytic activity collectively contributed to down-regulation of LOX by NNK in exposed cells. In view of LOX tumor suppressor activity and other biological functions, down-regulation of LOX by NNK is deeply involved in NNK pathogenesis and carcinogenesis.
